# Texas Training School for Defectives

**Published:** 1917-06

**Authors:** 


### Texas Training School for Defectives.

We wish to call the attention of the physicians who read this JOURNAL to the advertisement of the "Texas Training School for Defectives," which appears in this issue of the JOURNAL. We believe that many doctors will be glad to know that there is a place where they can send the backward children who are under their care, with the assurance to the parent that everything that is known to modern medical skill will be done for the unfortunate little one.

Dr. T. O. Maxwell, who is in charge of this home, has devoted many years to the sympathetic and individual study of defective children, and there is no person in the State or in the South for that matter who is better qualified to get the very best results than he.

Drs. Joe Wooten and A. F. Beverly are both trained experts in this work.

The institution is located upon one of the highest hills in Austin and commands a beautiful, restful outlook. It is a large commodious three-story brick with all modern conveniences that add to the health and comfort of the child. A large lawn with many large shade trees affords a delightful place for outdoor play, while the 2640 square feet of screened in galleries makes it possible to give the children the advantage of outdoor exercise the entire year. In fact, the institution combines school, home and sanitarium.

The well appointed school rooms are under the supervision of expert teachers who have broad experience with this class of children. The course includes the usual school work, manual training, care of the person, and correction of bad habits.

The school work is conducted in the same manner as any other well organized school. In the manual training work the children are taught to weave, make baskets, sew, darn, repair their clothing and to do many other useful things that will enable them to be self-supporting eventually.

The superintendent and resident physician reside in the institution and have direct supervision over the entire school. They are accessible at all times and gives such instruction and medical treatment, massage, special diet or any other attention they deem necessary.

The food is the best that the market affords and is prepared in an appetizing manner.

The children are treated in a kind and considerate way, and their defects are never the subject of remarks.

If you know of children who are needing the care of these trained experts, write Dr. Maxwell to send you one of the fully illustrated booklets, and show it to the parent of such a child. It will enable them to understand just how much the little one needs this special institution and how much can be gained by sending them before they are too old to receive and profit by the instruction.

